# The Use of a Non-Penetrating Captive Bolt for the Euthanasia of Neonate Piglets

**DOI:** 10.3390/ani8040048

**Published:** 2018-04-02

**Authors:** Andrew Grist, Jeff A. Lines, Toby G. Knowles, Charles W. Mason, Stephen B. Wotton

**Affiliations:** 1 School of Veterinary Sciences, University of Bristol, Langford House, Langford, Bristol BS40 5DU, UK; toby.knowles@bristol.ac.uk (T.G.K.); steve.wotton@bristol.ac.uk (S.B.W.); 2 Silsoe Livestock Systems, Wrest Park, Silsoe, Bedford MK45 4HR, UK; jeff.lines@silsoeresearch.org.uk; 3 Humane Slaughter Association, The Old School, Brewhouse Hill, Wheathampstead, Hertfordshire AL4 8AN, UK; charlie@hsa.org.uk

**Keywords:** animal welfare, euthanasia, livestock, mechanical killing, on-farm killing, neonate piglets

## Abstract

**Simple Summary:**

The humane destruction of newborn piglets (neonates), when required, is an issue faced by farmers and producers. The application of blunt force trauma, either through swinging the animal against a wall, or hitting it with a weighted object, is a stressful procedure for the stock person and has implications for the animal in terms of welfare, instantaneous effect and reproducibility. The United Kingdom government funded this project to find a single application method that could be used on farms that would produce an immediate kill with these animals. This project demonstrates that the use of a mechanical captive bolt device, that does not enter the head, delivers sufficient energy when applied to the head of a piglet to immediately destroy the brain leading to the death of the animal. This method will improve animal welfare on farms, as well as providing producers with a device that they can be confident will kill the animal without pain, as the brain is destroyed before the animal can perceive a pain nerve impulse.

**Abstract:**

The most common method for the on-farm euthanasia of neonate piglets is reported to be manual blunt force trauma. This paper presents the results of research to evaluate a mechanical non-penetrating captive bolt (the Accles and Shelvoke CASH small animal tool, Birmingham, UK) to produce an immediate stun/kill with neonate piglets. One hundred and forty-seven piglets (average dead weight = 1.20 kg ± 0.58 (standard deviation, SD), mean age = 5.8 days (median = 3)) were euthanized with the device and demonstrated immediate loss of consciousness, subjectively assessed by behavioural signs and no recovery. The result that 147 out of 147 animals were effectively stun/killed gives a 95% confidence interval for the true percentage of animals that would be effectively stun/killed of 97.5–100% with the use of the CASH small animal tool under the conditions of the current study. This research concludes that the CASH small animal tool, using a 1 grain brown coded cartridge, is suitable for producing a stun/kill in neonate piglets when applied in a frontal/parietal position.

## 1. Introduction

Occasionally, stockmen will be faced with the problem of having to dispatch, or euthanase, young piglets for various reasons including illnesses that are beyond treatment, birth deformations or production efficiencies. Usually, in the case of illness, the choices that are available to the producer are either leaving the neonates with their mothers in the hope that they may recover, or casualty slaughter. Euthanasia or slaughter of surplus young animals on-farm is usually carried out by administering a blow to the head, which is generally performed with a percussive blow or by swinging the young animal against the floor or a wall. Although widely used as a means of casualty slaughter, the effectiveness of this method is heavily dependent on the strength and skill of the operator and, consequently, the probability of consistently achieving an immediate kill is low. Furthermore, lack of proper training and human error can lead to pain and distress to the animal. It is also a method of killing that is aesthetically unpleasant for both the operator and any bystanders [1,2,3]. As the terms ‘dispatch’, ‘euthanasia’, ‘casualty or surplus slaughter’ and ‘culling’ are all commonly used to describe the termination of life, we will use the term euthanasia in this paper to encompass them all.

The Humane Slaughter Association carried out a survey in 1996 [4] to look at the euthanasia methods used for young lambs and piglets. The results showed that the majority of young, sick lambs are left to die, whilst a manual blow to the head was the normal method applied to casualty piglets. The majority of respondents were not satisfied with their current method of euthanasia and all of them expressed an interest in an alternative method. Unfortunately, currently there is no approved available alternative method for euthanizing young livestock in the United Kingdom.

There are many considerations for evaluating the effectiveness and acceptability of on-farm euthanasia techniques. Over the past several years, animal welfare experts, industry organizations, animal welfare groups and governmental agencies have detailed their primary concerns and recommendations in various publications [5,6,7,8,9,10]. While differing slightly, the basic criteria remain fairly constant: the ability to induce loss of consciousness and death without causing pain or distress, the time required for loss of consciousness and death, compatibility with intended animal use and purpose, operator safety, reliability, cost, practicality, aesthetics and emotional impacts, environmental impacts, and legal requirements.

A previous DEFRA project (United Kingdom Department for Environment, Food and Rural Affairs, MH0116) examined the reliability and output of several mechanical devices, one of which, the Accles and Shelvoke CASH Small Animal Tool (CPK200, Birmingham, UK) was found to be reliable, to produce a kinetic energy that would, theoretically, stun/kill neonates and which produced a consistent output. The use of an alternative tool, the Zephyr EXL for the euthanasia of piglets is reported elsewhere [11,12]. This paper examines the use of the Accles and Shelvoke Cash Small Animal Tool in practical use for the humane euthanasia of neonate piglets.

This paper reports the methods and findings of DEFRA project MH0150, “A study to investigate a non-penetrating percussive blow to the head as a humane killing method for piglets up to 5 kg”, a study approved by the University of Bristol’s Ethical Committee and carried out under a United Kingdom Home Office Licence (PPL 30/2999).

### Description of CASH Small Animal Tool

**Figure 1:**
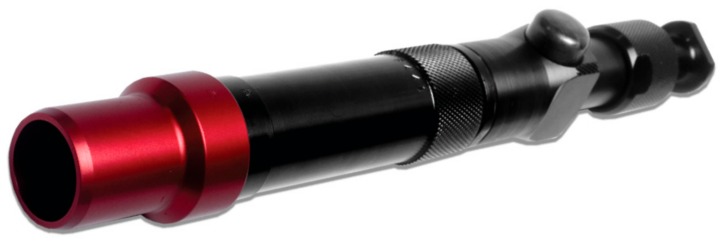
Accles and Shelvoke CASH Small Animal Tool. A 0.22” caliber blank cartridge-powered non-penetrating captive bolt device.

**Figure 2:**
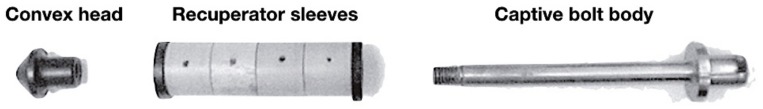
Bolt components of the non-penetrating captive bolt (adapted from Accles and Shelvoke).

The CASH Small animal tool, formally designated CPK200, is a non-penetrating captive bolt (Figure 1 and Figure 2) powered by a brown colour-coded 0.22” calibre rimfire blank cartridge containing 1 grain (65 mg) single base propellant (nitrocellulose) as its power source. The gas expansion chamber of the breech has a length of 20 mm. The bolt can travel further than 20 mm by disengaging from the expansion chamber. Although the total available travel for the bolt is 110 mm, it is constrained by buffers, or recuperating sleeves, that are slightly compressed even when the bolt is at rest and become progressively more compressed as the bolt extends. At rest, the bolt head is retracted 6–10 mm from the point at which it contacts the target. When the recuperating sleeves are in good condition the total travel of the bolt ranges from 25 mm to 35 mm. In a pure ballistic situation, the energy of the explosive cartridge is fully converted into kinetic energy before impact so that the sole source of energy at impact is the kinetic energy of the projectile. The CASH small animal tool cannot, therefore, be considered a ballistic device since the bolt may make contact with the target after a travel of only 5 mm or 10 mm, while it remains engaged with the expansion chamber. It is therefore subject to the force of the expanding gasses for a further 10–15 mm of travel. In the absence of an impact target, the bolt may be expected to accelerate over a distance of up to 20 mm and, thereafter, decelerate. Without a detailed knowledge of the velocity profile of the bolt as it impacts the target and the dynamic characteristics of the recuperating sleeves, it not possible to identify with precision the proportion of energy absorbed by the recuperating sleeves and that delivered to the target. However, the maximum velocity achieved by the bolt, in the absence of an impact target is likely to be a representative measure of the energy available at impact. The average kinetic energy produced on impact by this device is 47 Joules when using a 1 grain cartridge and 107 Joules when using a 1.25 grain cartridge. Previous work [11] demonstrated that a non-penetrating device producing a kinetic energy of 27 Joules was sufficient to stun/kill neonate piglets using loss of visual-evoked potentials and loss of brain stem reflexes as a determinant of brain death.

## 2. Materials and Methods

### 2.1. Animals and Procedures

The piglets (n = 202), average dead weight = 1.222 kg (± 0.665 (Standard Deviation, SD), mean age = 5.7 days (median = 3), used in this objective were commercial hybrid large white/landrace crosses bred by DanBreds International (Herlev, Denmark) and were animals destined to be euthanized according to the farm’s standard protocols either due to disease, malformation or production efficiency. All of the animals used in this project were either animals destined to be killed on the grounds of casualty slaughter or routine farm management during 18 visits to the commercial sow unit. The participating producers were instructed that animals that were suffering any pain and distress must not be held back and kept alive for this project but must be euthanased as soon as possible. Fifty-five piglets were euthanized with the device powered by a 1.25 grain cartridge. However, the cartridge strength chosen (pink 1.25-grain) resulted in damage to the piglets and excessive wear to the gun, in particular damage to the recuperator sleeves (buffers). Permission was obtained from the Home Office and DEFRA to lower the cartridge strength from 1.25-grain to 1-grain, following preliminary tests on cadavers, which reduced the average energy developed from 107 Joules to 47 Joules. The 1 grain powerload was used on the subsequent 147 piglets.

#### Device Application

**Figure 3:**
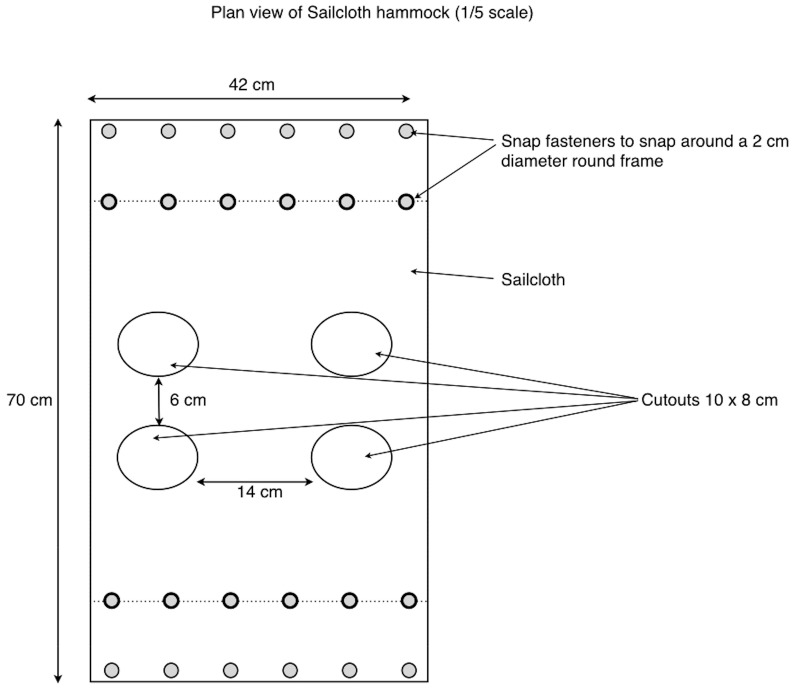
Sailcloth hammock design.

**Figure 4:**
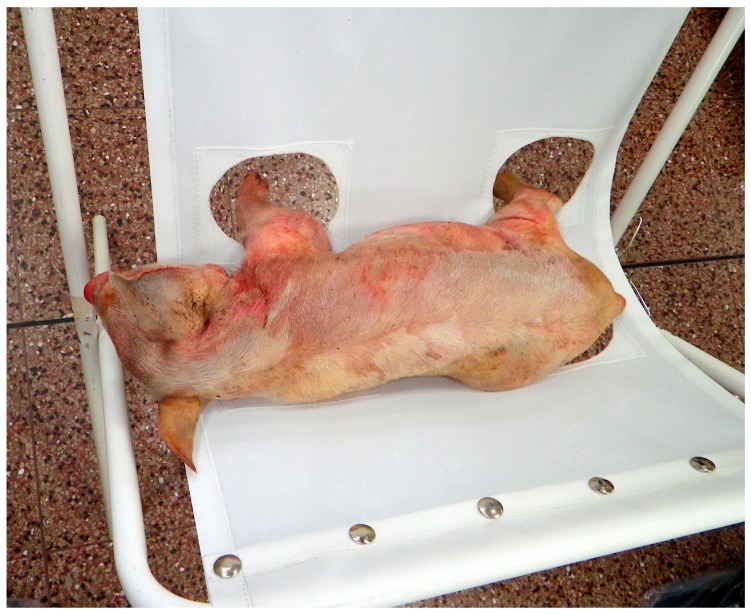
Sailcloth hammock in use (cadaver used to demonstrate restraint).

**Figure 5:**
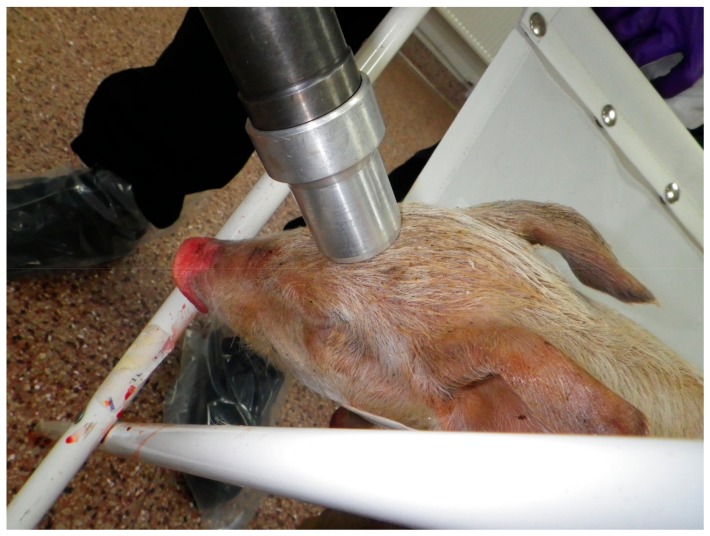
Shooting position for piglets on the midline on the frontal/parietal bone.

Piglets to be euthanized with the CASH small animal tool were placed in a sailcloth hammock attached to a tubular metal X-frame (Figure 3). This allowed the legs of the piglet to hang down without touching the floor and provided restraint for application of the device (Figure 4) and enabled a close evaluation of brain stem reflexes and piglet movement post-shot. The device was applied once in the frontal-parietal position of the piglet (Figure 5) by the researcher (Stephen B. Wotton) whilst gently restraining the animal with the free hand and assessed for behavioural signs of brain death. Previous research has shown that the following parameters meet the criteria for assessing the effectiveness of the application, i.e., clinical signs characterising a dysfunction affecting (i) the cerebral hemispheres on a large scale; (ii) the reticular formation; or (iii) the ascending reticular activating system or the median thalamus bilaterally [13]:

1. The absence of rhythmic breathing, which is controlled by structures within the medulla oblongata and innervated by the reticular activating system [13].
2. The absence of a positive corneal reflex, a reflex with a neural pathway that passes adjacent to, and partially through, the reticular formation [13].
3. The absence of a positive palpebral reflex, which is a brainstem reflex.
4. The absence of response to painful stimuli (needle prick to the nose), a cortical arc reflex [13].

Any animal that showed signs of recovery, or rhythmic breathing, within the 3-min evaluation time [14] or that persisted beyond the 3-min evaluation time, was killed immediately using an injected overdose of pentobarbital sodium (1 mL 200mg/mL injection into the heart), (Euthatal, Merial, UK, GTIN:03661103015550).

Following the shot, each piglet was examined by the researcher for behavioural signs of brain dysfunction. The presence of a heartbeat was also assessed by the researcher by auscultation, although this is not considered an indicator of death or life. Movement post-application (clonic activity, leg paddling) was assessed subjectively according to the descriptors in Table 1; time to end of movement was recorded from the application to the end of activity. All brain function measurements were continued for 3 min post-shot [14] and all findings were recorded by a technician.

### 2.2. Post Mortem Examination of Heads

#### 2.2.1. Section 1—Post Mortem Examination

All experimental animals were frozen after killing and subsequently thawed for post mortem examination. The intact heads of the piglets were photographed using a digital camera (PENTAX Optio WG-1 or PENTAX K-50 (Pentax Ricoh Imaging Company, Ltd, Tokyo, Japan). The skin from the head was removed following a T incision cranial to the shoulders and extending forward to the nose. The impact site was photographed before removal of any haematoma and the periosteum to expose fracture lines extending from the impact site. Photographs were taken of the fracture patterns to allow for later comparison. The heads were placed in individually numbered bags and subsequently hard frozen to facilitate sectioning on the sagittal plane for photography of cranial and brain lesions to be undertaken.

#### 2.2.2. Section 2—Sagittal Plane Assessment

Each head was removed from the bag once the number had been noted; the ear number was checked to ensure correlation. The head was split on the sagittal plane using an electric band saw (Startrite Meat Master, UK) and both sides were photographed on the medial plane with a digital camera (PENTAX Optio WG-1 or PENTAX K-50) and post mortem findings recorded.

**Figure 6:**
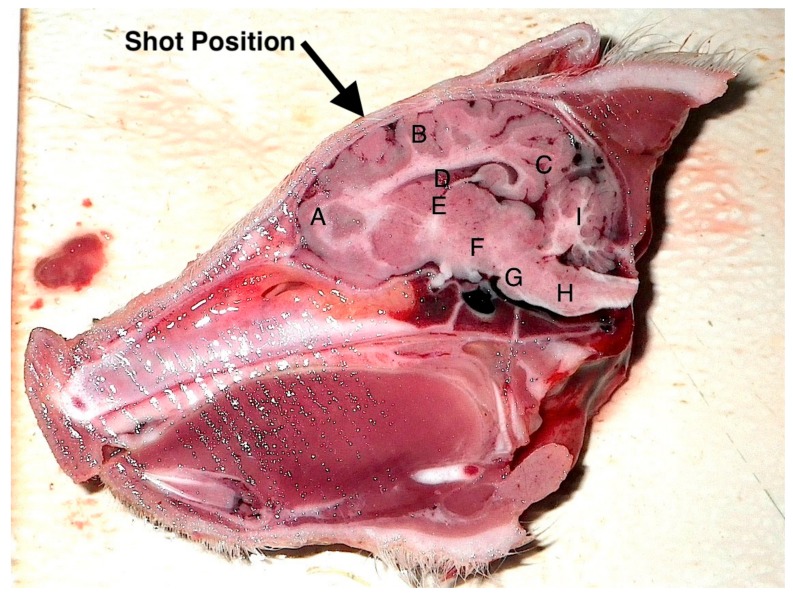
Sagittal section of an unshot piglet head (died on farm) illustrating shot position and the areas examined for macroscopic damage. A—frontal cerebrum, B—parietal cerebrum, C—occipital cerebrum, D—lateral ventricle. These were scored on the basis of 0 = no damage, 1 = slight deformation, 2 = moderate deformation and 3 = severe deformation of the area. Areas E–I (thalamus, midbrain, pons, medulla and cerebellum respectively) were assessed for the presence or absence of haemorrhage.

**Figure 7:**
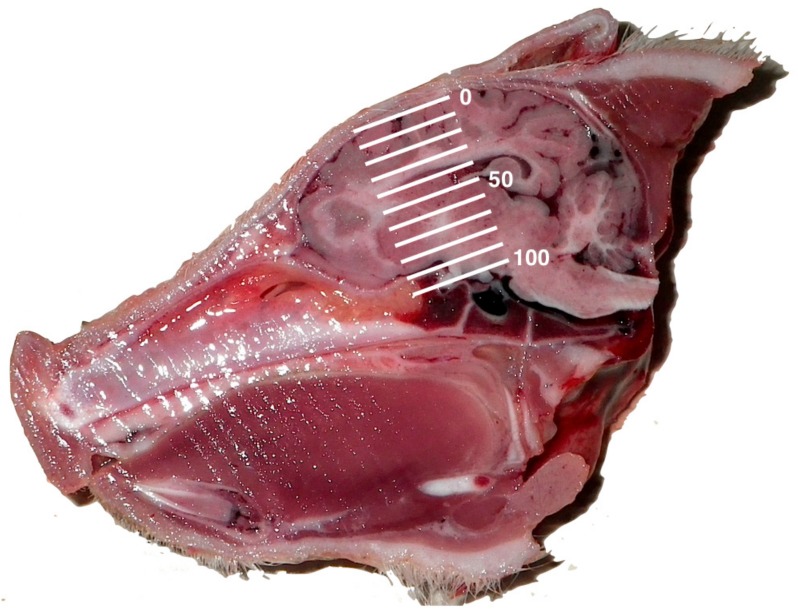
Skull-plate displacement diagram. The level of skull-plate displacement following the shot is expressed as a percentage, with 0 being no displacement and 100% being a case where the skull fragment was observed at the base of the cranial cavity. Each sagittal plane piglet photograph had the scale expanded proportionately for this assessment, with 0 located at the cranial surface and 100% located at the bone peak between the optic chiasma and mammillary body.

Two researchers assessed the macroscopic brain lesions separately, and blind to the weight and piglet number, utilising a subjective scale adapted from Sharp et al. [15] and reported in Grist et al. [12] where 0 = no damage, 1 = slight deformation, 2 = moderate deformation and 3 = severe deformation of the area. The researchers then agreed final figures. The areas examined for macroscopic damage were the frontal, parietal and occipital cerebrum including the structure of the lateral ventricle as detailed in Figure 6. The thalamus, midbrain, pons, medulla and cerebellum were assessed for presence or absence of haemorrhage. The percentage displacement of the cranial bone in relation to the surface was also assessed using a 0–100% scale that was expanded proportionately to each sagittal photograph, with 0% being the cranial surface and 100% located at the bone peak between the optic chiasma and mammillary body, such that a score of 50% would indicate that bone plates were evident half way between the normal cranial plate surface and the base of the brain (Figure 7).

### 2.3. Statistical Analysis

Statistical advice suggests that 100% efficiency can never be absolutely proven, there will always be some small margin for error, however large the study. However, a target sample size of 200 was in practice a reasonable figure to demonstrate the degree of efficacy of the method. A sample of this size would give a 95% confidence interval, should 100% of 200 animals be effectively stun/killed, that the very maximum possible percentage of animals not immediately stunned/killed in normal use, and therefore requiring a second shot, would be at most no more than 1.9% (Wilson’s Method [16]). In addition to presenting the confidence intervals for single sample estimates the correlations between relevant variables were assessed using Spearman’s Rho (r_s_). The postmortem macroscopic brain lesion results were also tested for an overall linear correlation with individual piglet weight and the score for each of the four areas using Spearman’s rank correlation test (Spearman’s Rho (r_s_)), in the IBM SPSS (v23) statistics package (SPSS Inc., Chicago, IL, USA). General linear models were also constructed to test for an effect of piglet dead weight, brain haemorrhage score, macroscopic brain damage score and nose/skin haemorrhaging/laceration on time to loss of movement, and also for their effect on movement score. The assumptions of normality of error and homogeneity of variance required for the models were tested and found to be satisfactory.

## 3. Results

The application of the CASH small animal tool resulted in an effective stun with every piglet and, in addition, every piglet was effectively killed by the procedure. Recovery of rhythmic breathing did not occur in these animals during the assessment period (3 min), which would indicate that severe, irrecoverable damage to the brain stem, the control centre for breathing, was achieved. The CASH small animal tool gun was initially used successfully on 55 piglets. A further 147 piglets were successfully stun/killed with the brown 1-grain cartridge, which resulted in reduced laceration to the skin over the impact area and less bleeding from the nostrils.

The result that 147 out of 147 animals were effectively stun/killed gives a 95% confidence interval for the true percentage of animals that would be effectively stun/killed of 97.5–100% with the use of the CASH small animal tool, with a 1-grain cartridge, under the conditions of the current study (Wilson’s Method [16]). The confidence interval for the 55 successfully killed using the 1.25 grain cartridge is 93.5–100%.

**Figure 8:**
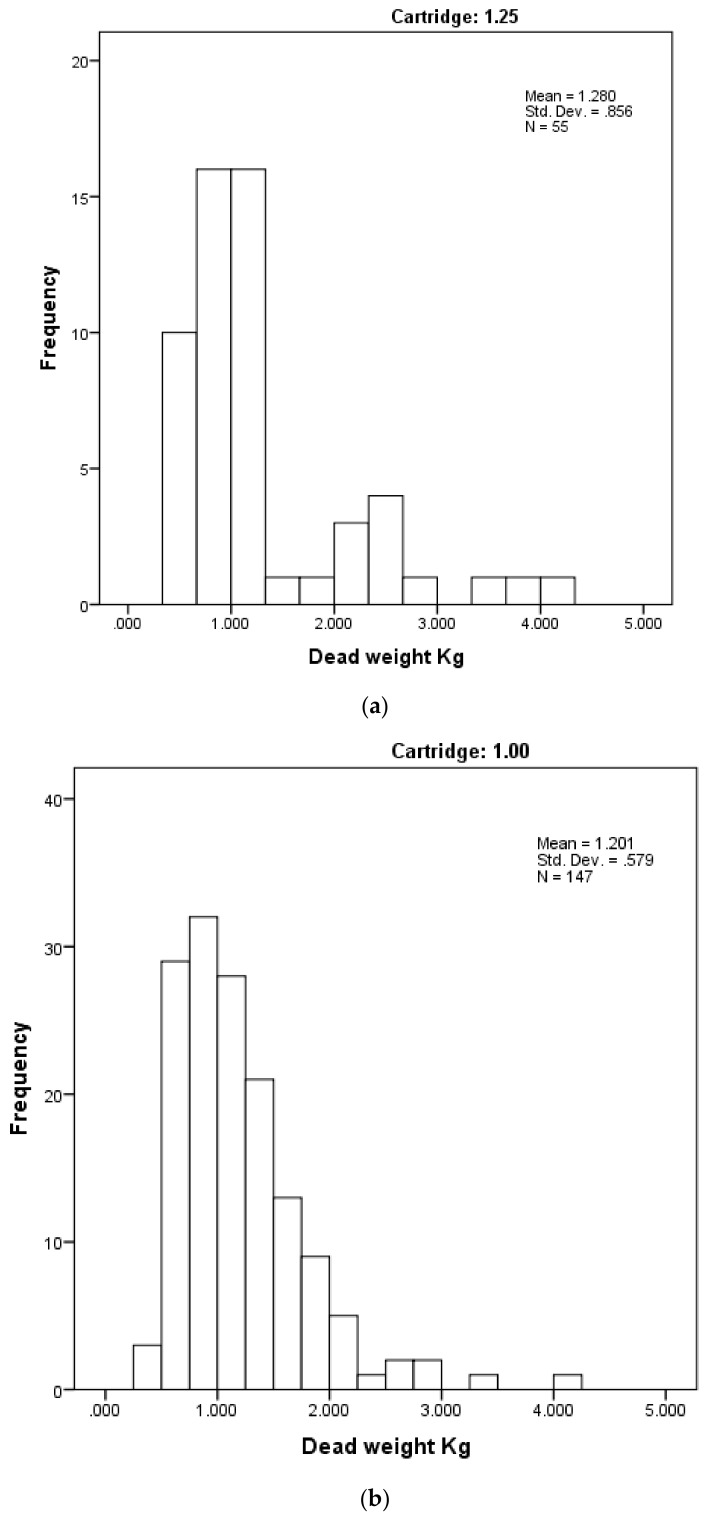
Distribution of piglet dead weights (kg) by cartridge used. (**a**) with 1.25 grain cartridge and (**b**) with 1 grain cartridge.

**Figure 9:**
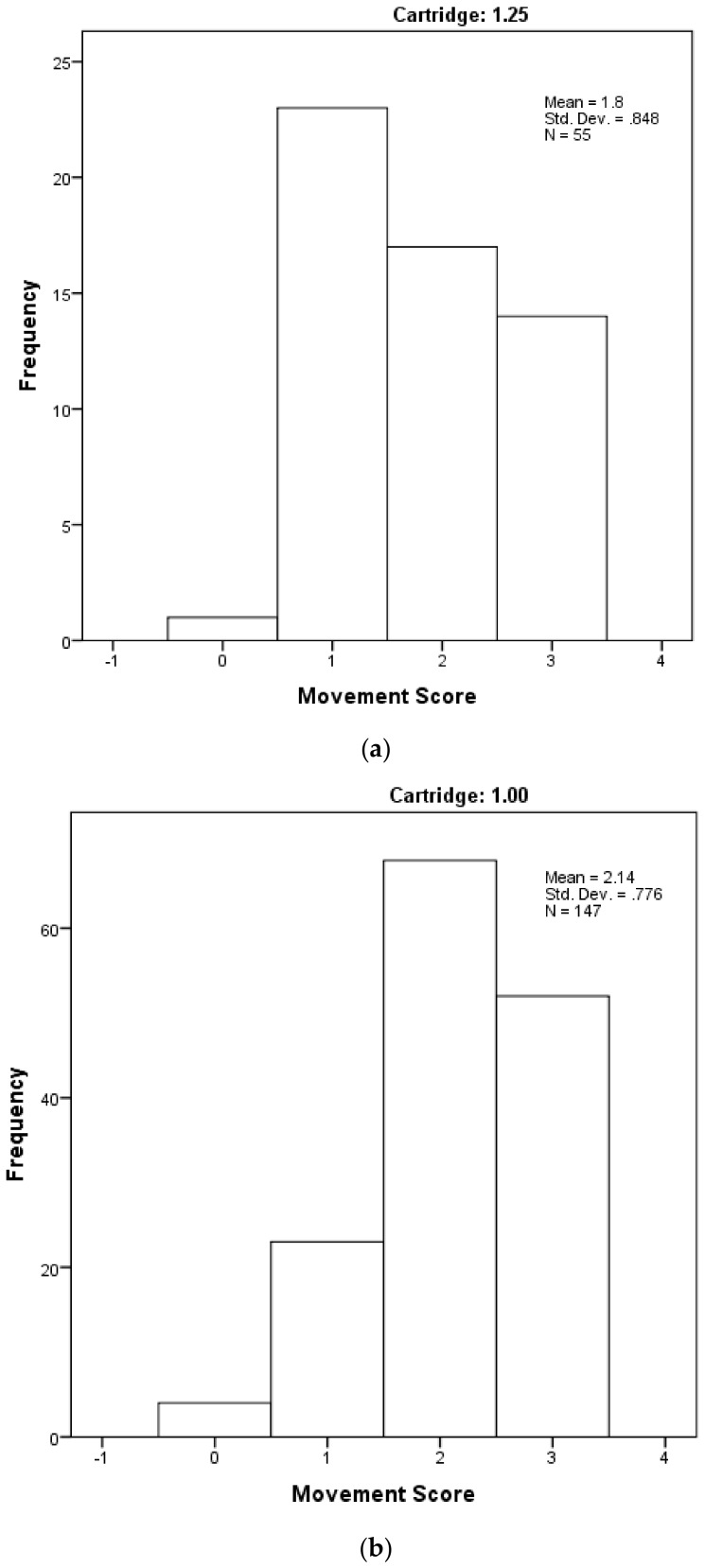
The distribution of piglet movement scores by cartridge-size used, based on a subjective scoring where Score 0 = very little movement; Score 1 = some mild uncontrolled physical movement of limbs; Score 2 = considerable uncontrolled physical movement of limbs; and Score 3 = gross uncontrolled physical movement. (**a**) with 1.25 grain cartridge and (**b**) with 1 grain cartridge.

Figure 8 shows the distribution of piglet dead weights (kg) across the range of casualty or surplus piglets that were presented for euthanasia by farm staff by the size of the cartridge used; and Figure 9 shows the frequency of the post-shot movement scores recorded. There was no significant difference in weight between the two groups (t = −0.754, *p* = 0.452), but a significant difference in movement score between the two groups (U = 3090, *p* = 0.006) with a mean score of 2.14 in the 1 grain cartridge group compared with 1.28 in the 1.25 grain group. The remaining variables were also tested for a difference between the two cartridge-size groups. The results are shown in Table 2 below.

The shard-plate displacement score correlated highly with the total brain damage score (R = 0.503, *p* < 0.001); therefore, in the following general linear model (GLM) analysis either one or the other was fitted; however, we include estimates for both in the tables of parameter estimates. The estimates for the remaining parameters are from the model when it included total score. This presentation makes no substantive difference to the interpretation of the results. The effect of cartridge-size group was also tested within the models as a main effect with the other variables present and with all possible 2-way interactions. All terms failed to reach significance in both the time to loss of movement model (all *p* > 0.40) and in the movement score model (all *p* > 0.410). Thus, cartridge group was dropped from both models.

### 3.1. Factors Related to ‘Time to Loss of Movement’

The parameter estimates from the GLM analysis of ‘time to loss of movement’ are shown in Table 3. This shows that there was no significant linear effect of dead weight, ‘total brain haemorrhaging score’, ‘total brain damage score’ or ‘shard plate displacement score’. There was a significant effect of the presence of nose/skin haemorrhaging/laceration on ‘time to loss of movement. On average, when these were present there was an associated decrease in ‘time to loss of movement’ of 36.86 s (standard error S.E. = 9.906,). The mean time to loss of movement for piglets without haemorrhaging (nose bleed or skin laceration) was 124.03 s (S.E. = 10.216) and the mean time to loss of movement for piglets with haemorrhaging (nose bleed or skin laceration) was 92.82 s (S.E. = 3.375). Note these are actual means, not marginal means estimated from the analysis.

### 3.2. Factors Related to ‘Movement Score’

**Figure 10:**
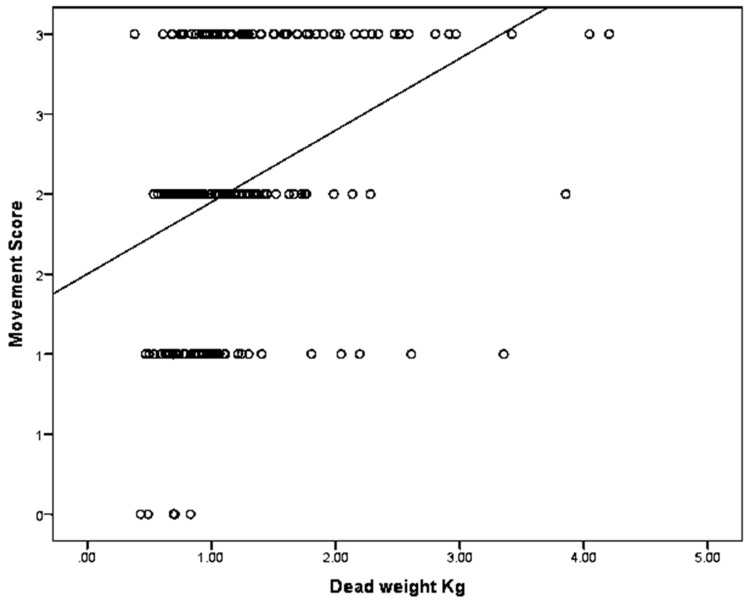
The relationship between piglet dead weight and movement score, where Score 0 = very little movement; Score 1 = some mild uncontrolled physical movement of limbs; Score 2 = considerable uncontrolled physical movement of limbs; and Score 3 = gross uncontrolled physical movement. N = 202.

The parameter estimates from the GLM analysis of ‘movement score’ are shown in Table 4. This shows that there was no significant linear effect of total brain ‘haemorrhaging score’, ‘total brain damage score’ or, ‘shard plate displacement score’, although there may appear to have been a trend for a relationship with total brain damage score. There was a significant effect of nose/skin haemorrhage/laceration presence on ‘movement score’ and the relationship between these is shown in Table 5. There was also a significant effect of piglet dead weight on the movement score and this is shown in Figure 10. The analysis suggests that for every 1 kg increase in piglet dead weight there was an associated average 0.39 (S.E. = 0.083) increase in movement score (*p* < 0.001).

### 3.3. Agonal Breathing

Eight of the 202 (4%) piglets developed agonal breathing, *n* = 7, >3 min post-shot and *n* = 1, ≤3 min post shot. All eight were in the 1-grain cartridge group. Post mortem findings suggest that the piglets that displayed agonal breathing suffered such severe brain damage that they could not have recovered from the percussive blow.

The mean duration of convulsions was 1 min 43 s and depended largely on the physical state of the animal when it was dispatched. Some casualty piglets were in a very poor physical condition and these animals tended to move less post-shot.

### 3.4. Post Mortem

**Figure 11:**
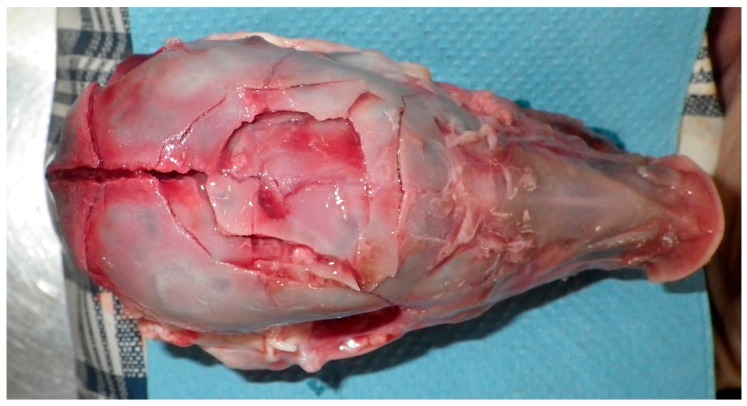
Fracture pattern at impact point. Skin, periosteum and haematoma removed.

All animals displayed a depressed fracture of the cranial plates and concurrent subdural haematoma corresponding to the impact footprint (Figure 11) of the convex head of the bolt (Figure 2). Fracture of the parietal plate was a common finding with bone shards forced into the medial dorsal cerebrum resulting in crushing of the parietal lobe of the cerebrum.

**Figure 12:**
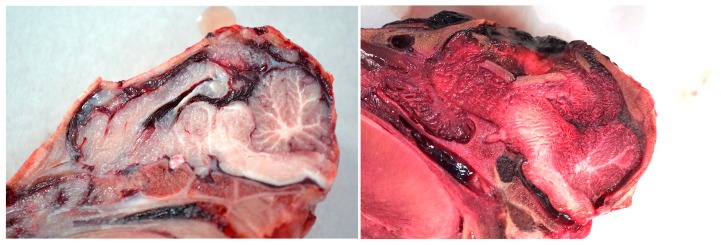
Examples of post-shot trauma; medial sagittal section demonstrating bone fracture displacement and macroscopic brain damage (1 grain cartridge).

The structure of the corpus callosum was generally severely compromised and parenchymal ecchymosis evident within the thalamus, frontal parietal and occipital lobes of cerebrum and the cerebellum (Figure 6 and Figure 12).

### 3.5. Haematoma and Nasal Haemorrhages

Table 6 shows the percentage and numbers of piglets that displayed nose bleed, broken skin and both, by cartridge size. A chi-square test showed there to be a highly significant association between these conditions and the size of cartridge used.

### 3.6. Cranial Bone Displacement

There was no significant correlation between piglet weight and the percentage bone displacement.

## 4. Discussion

This research demonstrated that the use of this percussive stun/kill device with a 1-grain cartridge resulted in an effective stun/kill with neonate piglets less than 28 days old (mean dead weight = 1.20 kg (±0.58 (SD)). The result that 147 out of 147 animals were effectively stun/killed using a 1-grain cartridge gives a 95% confidence interval for the true percentage of animals that would be effectively stun/killed of 97.5–100% with the use of the CASH small animal tool (CPK200) under the conditions of the current study (Wilson’s method [16]), and a 95% confidence interval of 93.7–100% for the 57 killed using the 1.25 grain cartridge. The CPK200 should be powered by a 1 grain cartridge, applied on the midline on the frontal/parietal bone. Although a 1.25 grain cartridge would also be successful, it is standard operating procedure that a cartridge that delivers more than the required power is not used as it shortens the life expectancy of the device. In addition, the higher grain cartridge produced less nasal hemorrhaging and more laceration at the application position.

### 4.1. Post-Shot Movement

Animal movement post-shot, i.e., clonic convulsions, are an expected result of an effective mechanical stun [17,18]. However, the presence of any movement, whether convulsions or agonal breathing following euthanasia on-farm, is aesthetically unpleasant for both the operator and any bystanders. Ideally an effective stun/kill would produce an immobile animal, but this is not the case in practice. The increase in the amount of post-shot movement that was associated with an increase in dead weight is likely to be related to the physical state of the casualty animals when they were shot. Smaller animals were less physically fit than larger piglets. The statistically significant longer movement times of piglets with no haemorrhage post-shot is an expected result, given that there is still an active heartbeat; the blood pressure reduction in those that bled either through laceration or nasal haemorrhage would exacerbate the reduction in available oxygen for muscle movement in conjunction with the lack of rhythmic breathing due to brain stem death. Similar research reported by Casey-Trott et al. [19,20] found that the average duration of convulsion was 3.8 min and 3.4 min, respectively, in neonatal pigs killed using a non-penetrating captive bolt. The onset of convulsions has been associated with the onset of an isoelectric electroencephalogram (EEG) and is one of the symptoms of an effective stun [21,22]. Gibson et al. [23] also suggested that an isoelectric EEG was incompatible with awareness. In addition, convulsions occur when modulation of the descending somatomotor activity from the brain, by the somatomotor cortex, is absent. Results from Terlouw et al. [18] show that paddling and neck movements can be observed in stunned, unconscious cattle even if the spinal cord and brain are no longer connected. Therefore, the presence of convulsions, while unsettling to the operator, could potentially be a useful indicator of an effective stun and loss of residual consciousness. Additionally, the presence of these convulsions and/or loss of muscle tone could potentially be used as indicators of early brain failure.

### 4.2. Agonal Breathing

Agonal breathing, or gasping respiration in the dying animal, is the last respiratory pattern prior to terminal apnoea. The duration of the gasping respiration phase varies; it may be as brief as one or two breaths, or as a prolonged period of gasping lasting minutes or even hours. Gasping respiration is very abnormal, easy to recognise and distinguish from other respiratory patterns and, in the dying animal, will always result in terminal apnoea [23]. St John [24] states that agonal or intermittent gasping can be induced by ischaemia or hypoxia and demonstrates dysfunction of brain centres in the pons and is due to medullary mechanisms. In a previous study [11] nine (15%) of the piglets shot with a percussive gun (Zephyr EXL, Bock Industries, Philipsburg, PA, USA) demonstrated agonal gasping >3 min post-shot. These piglets were anaesthetized and visual evoked potentials (VEPs) recorded pre-, and post-shot. The loss of VEPs and the isoelectric EEG following application from these animals confirmed the absence of cortical brain activity.

### 4.3. Brain Damage

The damage to the cerebrum and cerebellum (shard-plate displacement scores), and the haemorrhage within brain structures point to the action of impact pressure waves and physical trauma to the brain that would be inconsistent with normal cortical function. Terlouw et al. [13] discusses the loss of consciousness to be associated with damage to either one or more of the cerebral hemispheres, the reticular formation or the ascending reticular activating system, or the median thalamus bilaterally. The degree of trauma produced was considered sufficient to produce immediate loss of consciousness and death, a finding that has also been demonstrated elsewhere with piglets [11]. The initial concern that skull structure in neonate piglets would be insufficient to produce differential acceleration was not demonstrated due to the level of physical trauma produced by the blow.

### 4.4. Incidental Brain Lesions

**Figure 13:**
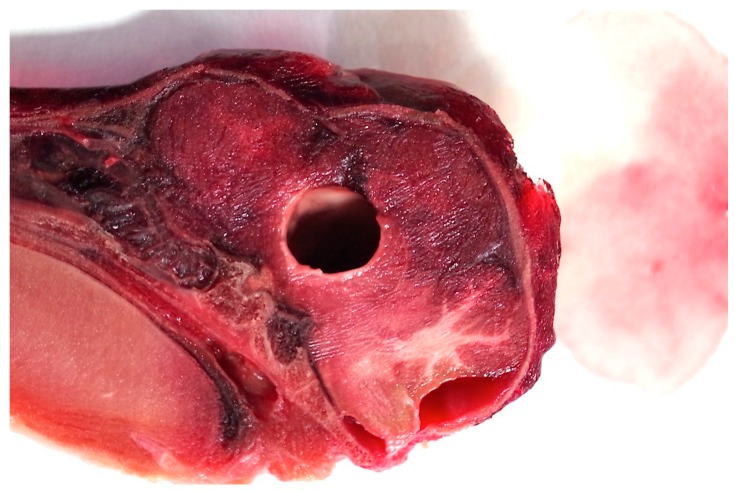
Neopallial cyst (proencephaly) within the brain of piglet 171 (sagittal section).

An incidental finding during the post mortem examination of the heads was that six animals (2.97%) displayed cerebral lesions, two were consistent with proencephaly within the cerebrum resulting from lack of development or cell destruction within the cortex, occasionally due to viral infections in utero [25]. These cysts are usually surrounded by a membrane of astroglial cells that appears white (Figure 13). Two further animals displayed lesions consistent with abscess formation.

## 5. Conclusions

Mechanical blunt force trauma provided by the Accles and Shelvoke Small Animal Tool using a 1 grain cartridge provides an immediate, reproducible, single-shot, stun/kill of neonate piglets that may require euthanasia for reasons including disease control or production efficiencies. This immediacy of action and reproducibility improves the welfare of these animals. It is important that the behavioural signs of a proper stun/kill application are explained to operatives. It is concluded that the use of the CASH small animal tool (CPK 200), percussive stun/kill device can be recommended for neonate piglets when a shot position on the midline on the frontal/parietal bone is used together with a 1 grain cartridge. A higher grain cartridge (1.25 grain) was also found to be effective but is not recommended for general use as this causes excessive wear to gun components, in particular the recuperator sleeve (buffers).

## Figures and Tables

**Table 1:** Subjective scoring system used to assess post-stun/kill movement based on the level of spinal reflex activity, ranging from 0 (no clonic activity post-stun) to 3 (severe uncontrolled physical movement).

Score	Descriptor	Description of intensity
0	No activity	Very little clonic activity.
1	Mild activity	Some mild uncontrolled physical movement of limbs.
2	Moderate activity	Considerable uncontrolled physical movement of the limbs.
3	Severe	Severe uncontrolled physical convulsive movement, paddling of legs

**Table 2:** Results of the tests for differences in the remaining outcome variables between the two cartridge sizes, showing the U statistic, significance and mean (standard error, S.E.) within each group.

Parameter	U	Exact *p*	1-grain		1.25-grain	
			Mean	S.E.	Mean	S.E.
Time to loss of movement (s)	3760	0.446	101.88	4.49	86.95	4.21
Total Haemorrhage Score	2522.5	0.014	4.63	0.57	4.32	0.13
Total Damage Score	1543.5	0.000	7.22	0.14	8.68	0.26
Plate or Shard %	1215	0.000	15.2	0.97	27.44	1.78
Presence of nose bleed	3370.5	0.009	85.70%	2.30%	7.80%	6.50%

**Table 3:** Parameter estimates from the general linear model (GLM) testing for an effect of dead weight (kg), brain haemorrhage score, macroscopic brain damage score, and nose/skin haemorrhaging/laceration on time to loss of movement.

Parameter	B	S.E.	*t*	Sig.
Intercept	134.031	27.562	4.863	0.000
Dead weight (kg)	−1.935	5.628	−0.344	0.731
Total brain haemorrhage score	4.930	4.919	1.002	0.317
Total brain damage score	−3.270	2.023	−1.616	0.108
Nose/skin haemorrhage/laceration	−36.856	9.906	−3.721	0.000
Shard-plate displacement score	−0.412	0.280	−1.473	0.142

**Table 4:** Parameter estimates from the GLM testing for an effect of dead weight (kg), brain haemorrhage score, macroscopic brain damage score, and nose/skin haemorrhage/laceration on movement score.

Parameter	B	S.E.	*t*	Sig.
Intercept	1.491	0.408	3.651	0.000
Dead weight (kg)	0.385	0.083	4.622	0.000
Nose/skin haemorrhage/laceration	0.488	0.147	3.325	0.001
Total brain haemorrhage score	0.024	0.073	0.324	0.746
Total brain damage score	−0.050	0.030	−1.656	0.099
Shard-plate displacement Score	−0.003	0.004	−0.752	0.453

**Table 5:** The number of piglets that demonstrated a nose bleed or skin laceration for each movement score, where Score 0 = very little movement; Score 1 = some mild uncontrolled physical movement of limbs; score 2 = considerable uncontrolled physical movement of limbs; and Score 3 = gross uncontrolled physical movement.

Nose bleed or Skin laceration	Movement Score			
	0	1	2	3
Yes	1	26	77	60
No	4	20	8	6

**Table 6:** The percentage of piglets with nose bleed and broken skin by cartridge size. Actual numbers are in brackets. There was a highly significant association between grain size and condition (chi-square = 70.03, df = 3, exact *p* < 0.001). Analysis of the effect of haemorrhages from the nasal passages and skin lacerations is given in Table 4 and Table 5.

Cartridge Size	None	Nose Bleed (Nb)	Skin Broken (Sb)	Nb and Sb
1-grain	14.3% (21)	63.9% (94)	2.7% (4)	19.0% (28)
1.25-grain	30.9% (17)	20.0% (11)	41.8% (23)	7.3% (4)
Total	18.8% (38)	52.0% (105)	13.4% (27)	15.8% (32)
